# Proximal Jejunal Bypass with Sleeve Gastrectomy for Severe Obesity and Type 2 Diabetes: First Case Report in Japan

**DOI:** 10.70352/scrj.cr.25-0392

**Published:** 2025-10-17

**Authors:** Taiki Nabekura, Takashi Oshiro, Kotaro Wakamatsu

**Affiliations:** 1 Department of Surgery, Toho University Sakura Medical Center, Sakura, Chiba, Japan; 2 Department of Surgery, The Jikei University, Tokyo, Japan

**Keywords:** bariatric surgery, metabolic surgery, jejunum, bypass surgery, diabetes, obesity

## Abstract

**INTRODUCTION:**

Laparoscopic sleeve gastrectomy is the most common metabolic bariatric surgery performed in Japan. Nevertheless, concerns persist regarding its long-term efficacy, which is considered inferior to that of procedures involving gastrointestinal bypass. In response to these concerns, a modified approach known as the “sleeve plus procedure” has been introduced and is now covered by insurance for patients with severe obesity and type 2 diabetes mellitus (T2DM). We successfully performed proximal jejunal bypass with sleeve gastrectomy (PJB-SG), a variant of this approach, marking the first documented case of its kind in Japan. This report presents the clinical outcomes of this procedure along with a review of the relevant literature.

**CASE PRESENTATION:**

A 50-year-old female patient with a body mass index of 46.9 kg/m^2^ presented with obstructive sleep apnea and T2DM. The patient exhibited resistance to pharmacological treatment, including glucagon-like peptide-1 receptor agonists and sodium-glucose cotransporter 2 (SGLT2) inhibitors. Fifteen months after the initial consultation, she opted for PJB-SG. The operation lasted 207 min, with 18 mL of blood loss. At 252 days postoperatively, the patient had achieved a total weight loss of 18.0% and a hemoglobin A1c level of 6.0% while continuing a minimal dose of SGLT2 inhibitors. No adverse events related to the bypass procedure, such as diarrhea or liver dysfunction, were observed.

**CONCLUSIONS:**

To our knowledge, this is the first reported case of PJB-SG conducted in Japan. This procedure may represent a promising alternative for patients with severe obesity and T2DM in Japan.

## Abbreviations

BEA-ERCP
: balloon enteroscopy–assisted ERCP
BMI
: body mass index
CPAP
: continuous positive airway pressure
DJB-SG
: duodenojejunal bypass with sleeve gastrectomy
ERCP
: endoscopic retrograde cholangiopancreatography
HbA1c
: hemoglobin A1c
JIB
: jejunoileal bypass
LA-ERCP
: laparoscopy-assisted ERCP
MBS
: metabolic bariatric surgery
PJB
: proximal jejunal bypass
PJB-SG
: PJB with sleeve gastrectomy
RYGB
: Roux-en-Y gastric bypass
SAS
: sleep apnea syndrome
SFA
: subcutaneous fat area
SG
: sleeve gastrectomy
SGJB
: sleeve gastrectomy with jejunal bypass
SG + JJB
: SG plus jejunojejunal bypass
SGLT2
: sodium-glucose cotransporter 2
T2DM
: type 2 diabetes mellitus
VFA
: visceral fat area
%TWL
: percentage total weight loss

## INTRODUCTION

The impact of metabolic bariatric surgery (MBS) on weight loss and the improvement of obesity-related comorbidities, including type 2 diabetes mellitus (T2DM), in Japanese patients with severe obesity has been reported in previous studies.^1)^ In Japan, sleeve gastrectomy (SG) has been the dominant procedure, accounting for more than 90% of annual cases,^2)^ mainly due to its coverage under the national health insurance system and its relative simplicity with a lower risk of complications compared to bypass procedures. However, SG has been associated with greater weight regain and worse glycemic control after surgery compared to bypass procedures.^3)^

Since June 2024, various bypass procedures combined with SG, the so-called “sleeve plus procedures,” have been included under Japanese national health insurance coverage for patients with T2DM and obesity. This is expected to increase the use of bypass surgeries in Japan. Among the sleeve-plus procedures, we adopted the proximal jejunal bypass (PJB) with SG (PJB-SG), originally reported by Alamo et al.^4,5)^ for its balance of safety and efficacy. PJB-SG is designed to optimize metabolic benefits while reducing the risks associated with more complex bypass procedures. One such example is duodenojejunal bypass with SG (DJB-SG), which involves duodenal dissection and 2 anastomoses (duodenojejunostomy and jejunojejunostomy).

This report presents the first documented case of PJB-SG performed on a patient with severe obesity and T2DM in Japan.

## CASE PRESENTATION

The patient is a 50-year-old female who presented with severe obesity and a history of repeated weight regain.

A review of her developmental history revealed no significant abnormalities. In her 40s, she began gaining weight and was diagnosed with sleep apnea syndrome (SAS), for which she initiated continuous positive airway pressure (CPAP) therapy. Despite good adherence to nutritional therapy, she was unable to maintain weight loss, resulting in her referral to our hospital.

At the initial visit, her height, weight, and body mass index (BMI) were 156 cm, 114.2 kg, and 46.9 kg/m^2^, respectively. The Eastern Cooperative Oncology Group Performance Status score was 0. She had SAS and T2DM with a modified ABCD (age, BMI, serum C-peptide, and duration of diabetes) score of 8. She also had comorbidities, including allergic rhinitis and depression.

She reported occasional alcohol consumption and denied smoking.

Her family history was notable for myocardial infarction, epilepsy, and alcoholism in her father; chronic obstructive pulmonary disease, bronchial asthma, and angina in her mother; and bipolar disorder in both her younger sister and elder daughter.

Her medication regimen included dapagliflozin (10 mg/day), along with other medications specified in **Table 1**.

**Table 1:** Medications at the initial visit

Vonoprazan fumarate	10 mg/day
Dapagliflozin propylene glycolate hydrate	10 mg/day
Rebamipide	300 mg/day
Magnesium oxide	990 mg/day
Pemafibrate	0.4 mg/day
Lemborexant	10 mg/day
Trazodone hydrochloride	100 mg/day
Venlafaxine hydrochloride	75 mg/day
Eszopiclone	2 mg/day

The patient exhibited a satisfactory hemoglobin A1c (HbA1c) level of 6.6%. Liver and kidney function tests were within normal limits, and no vitamin deficiencies were observed (**Table 2**).

**Table 2:** Blood examination results at the initial visit

C-reactive protein	0.25 mg/dL
Total protein	7.9 g/dL
Albumin	4.3 g/dL
AST	60 U/L
ALT	122 U/L
LDH	180 U/L
ALP	98 U/L
GGT	61 U/L
Total bilirubin	0.5 mg/dL
BUN	12.2 mg/dL
Creatinine	0.44 mg/dL
Sodium	138 mEq/L
Potassium	4 mEq/L
Chloride	103 mEq/L
Calcium	10 mg/dL
Inorganic phosphorus	3.7 mg/dL
Iron	81 μg/dL
Total cholesterol	210 mg/dL
Triglyceride	199 mg/dL
HDL cholesterol	50 mg/dL
LDL cholesterol	132 mg/dL
Zinc	88 μg/dL
Fasting blood glucose	128 mg/dL
Vitamin B1	3.7 μg/dL
Vitamin B12	579 pg/mL
Folic acid	8.3 ng/mL
25-Hydroxyvitamin D3	15.3 ng/mL
HbA1c	6.6%
White blood cell count	8800/μL
Red blood cell count	466 × 10^4^/μL
Hemoglobin	13.8 g/dL
Hematocrit	43.1%
Platelet count	25.7 × 10^5^/μL

ALP, alkaline phosphatase; ALT, alanine aminotransferase; AST, aspartate aminotransferase; BUN, blood urea nitrogen; GGT, gamma-glutamyl transferase; HbA1c, hemoglobin A1c; HDL, high-density lipoprotein; LDH, lactate dehydrogenase; LDL, low-density lipoprotein

Preliminary assessments, including respiratory function, electrocardiographic, and echocardiographic results, revealed no abnormalities.

Upper gastrointestinal endoscopy revealed superficial gastritis. No esophagitis or hiatal hernia was detected. *Helicobacter pylori* antibodies were detected in the urine, and the patient was preoperatively treated for its eradication.

CT revealed fatty liver with no additional significant findings. The visceral fat area (VFA) was 392.2 cm^2^, and the subcutaneous fat area (SFA) was 665.5 cm^2^. The VFA/SFA ratio was 0.59.

### Course of medical treatment and processes leading to surgery

Glucagon-like peptide-1 receptor agonist therapy was initiated; however, severe digestive side effects led to poor tolerability and necessitated its discontinuation. Consequently, sodium-glucose cotransporter 2 (SGLT2) inhibitor therapy with dapagliflozin tablets was initiated and titrated to a dose of 10 mg. Despite these pharmacological interventions, the patient continued to experience inadequate weight loss, which led to the consideration of surgical intervention as a potential solution. Following a multidisciplinary team conference at our hospital, a consensus was reached to proceed with MBS to achieve weight reduction and metabolic improvement. The patient underwent the surgical procedure 15 months after the initial consultation, at which point her weight was 112.1 kg, reflecting a 2.1 kg (percentage total weight loss [%TWL], 1.8%) reduction from baseline.

### Surgical technique

**Fig. 1:**
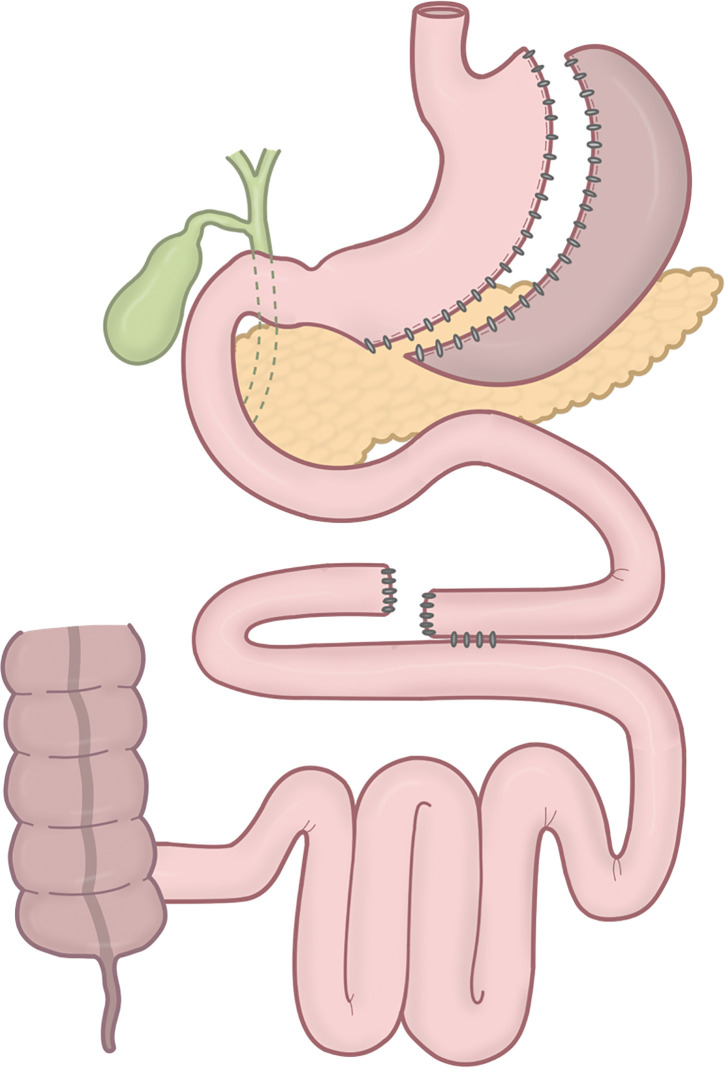
A schematic of proximal jejunal bypass with sleeve gastrectomy. Following sleeve gastrectomy, the small intestine is transected 150 cm distal to the ligament of Treitz, and a side-to-side jejunoileal anastomosis is created 250 cm proximal to the ileocecal valve, thereby excluding a segment of the small intestine from the alimentary tract.

**Fig. 2:**
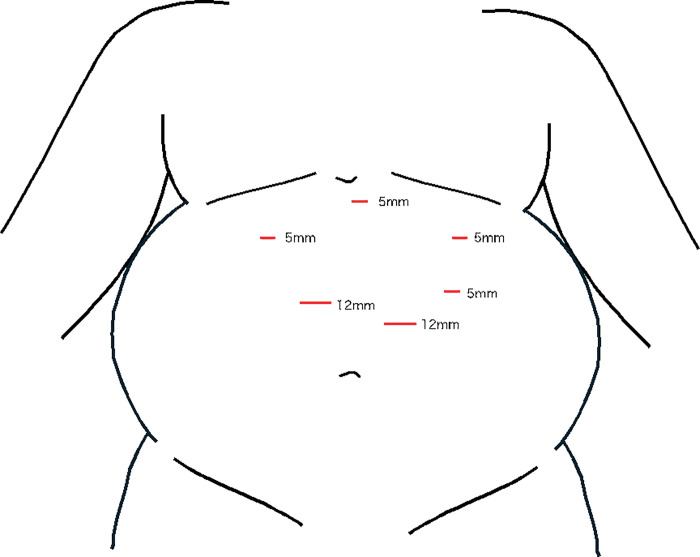
Surgical port placement.

**Figure 1** shows a schematic of the procedure. The patient was placed in the lithotomy position, with the right arm tucked after induction of general anesthesia. The procedure was performed laparoscopically using 6 ports. The port placement is shown in **Fig. 2**. A 36-Fr calibration bougie tube (laparoscopic sleeve gastrectomy tube; SB Kawasumi, Kawasaki, Kanagawa, Japan) was inserted orally. The lateral segment of the liver was elevated using a silicone disk (Hakko, Chikuma, Nagano, Japan) and a retractor (Diamond-Flex circle retractor; Niti-On, Funabashi, Chiba, Japan), providing a clear view of the esophagogastric junction. The greater omentum was dissected to expose the left crus of the diaphragm using an advanced bipolar device (LigaSure Maryland 44 NC; Medtronic, Minneapolis, MN, USA). Resection was started orally, 5 cm from the pylorus along the greater curvature, with the final transection point 1 cm distal to the angle of His. A sleeve-shaped resection was completed along the oral calibration tube using 6 cartridges of staplers (Endo GIA with Tri-Staple technology and Signia stapling system; Medtronic), with a reinforced reload on the final shot. The staple line was reinforced with seromuscular and full-thickness suturing using nonabsorbable 3-0 barbed suture (V-Loc PBT wound closure device; Medtronic) to ensure hemostasis. To prevent twisting or thoracic migration of the remnant stomach, it was fixed to the transverse mesocolon. The proximal end of the staple line was covered with a polyglycolic acid sheet (Neoveil; Gunze, Ayabe, Kyoto, Japan).^6)^

**Fig. 3:**
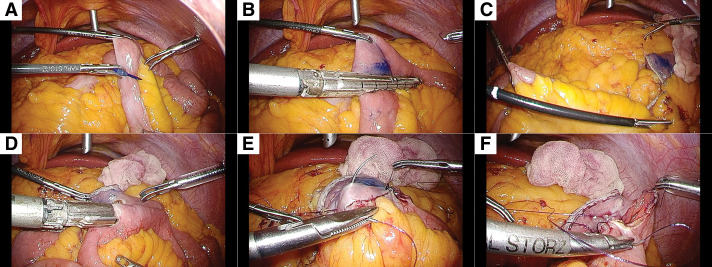
Intraoperative findings. (**A**) Marking the point 150 cm distal from the ligament of Treitz with a surgical pen. (**B**) The small intestine is transected with a stapler parallel to the mesentery. (**C**) Counting 250 cm proximal to the ileocecal valve. (**D**) Side-to-side anastomosis is performed using a 45-mm stapler. (**E**) The staple line is continuously sutured with a 3-0 monofilament absorbable thread. (**F**) The common entry hole is closed with continuous suturing using a 3-0 monofilament absorbable thread.

After completing the SG, the patient was placed in a slight head-down position. The greater omentum and transverse colon were lifted cranially to expose the ligament of Treitz. The small intestine was transected 150 cm distal to the ligament of Treitz (**Fig. 3A** and **3B**). A distance of 250 cm was measured proximally from the ileocecal valve (**Fig. 3C**), and an anastomosis was planned at this site with the proximal intestinal stump. The length of the bypassed segment was confirmed to be 300 cm. A small window was created on the antimesenteric side, and a side-to-side anastomosis was performed using a 45-mm Endo GIA stapler (Medtronic) in a reverse peristaltic manner (**Fig. 3D**). To ensure hemostasis at the anastomosis, the staple line was circumferentially sutured with 3-0 monofilament absorbable thread (Monocryl Plus Antibacterial suture; Ethicon, Blue Ash, OH, USA) (**Fig. 3E**). The entry hole was then closed with continuous suturing using 3-0 monofilament absorbable thread, after confirming hemostasis and the absence of stenosis of the lumen (**Fig. 3F**). The stump of the bypassed jejunum was also sutured using 3-0 monofilament absorbable thread to achieve hemostasis. The mesenteric defect was closed using 3-0 nonabsorbable barbed sutures.

The total operative time was 207 min, and blood loss was 18 mL.

### Perioperative and postoperative course

The patient was managed in a general ward after surgery, and CPAP therapy was resumed on the 1st postoperative night. The patient ambulated and started a liquid diet on POD 1. On day 2, an upper gastrointestinal series was performed, confirming that there were no problems with the shape of the stomach sleeve or leakage at staple lines. Diabetes medications were discontinued, and the patient was discharged on POD 5 at a weight of 109.5 kg.

At 252 days after surgery, her weight had decreased to 93.6 kg, representing an 18.0 %TWL. The patient’s HbA1c level was 6.0%, and she was maintained on 5 mg of dapagliflozin. She did not exhibit signs or symptoms indicative of persistent diarrhea, liver dysfunction, or nutritional deficiency. Follow-up blood tests conducted over time have consistently yielded normal results, with levels of albumin, hemoglobin, iron, vitamin B12, and fat-soluble vitamins remaining within the standard range. Moving forward, we intend to monitor the patient’s nutritional status every 3 months. Standard multivitamin supplementation will be recommended, as is customary in patients undergoing bypass procedures.

## DISCUSSION

Recent reports suggest that MBS, including bypass procedures such as Roux-en-Y gastric bypass (RYGB), is more effective in the long term for weight loss and improvement of comorbidities in patients with severe obesity than SG alone.^7)^ Conversely, other studies have reported no significant differences between SG and bypass procedures,^8)^ thereby complicating the determination of the optimal surgical approach. The choice of specific surgical technique depends on the patient’s condition and the surgeon’s expertise in performing the respective procedure.

RYGB remains the most prevalent bariatric procedure in Western countries. However, its application in Japan is limited due to the high incidence of gastric cancer, largely attributed to *Helicobacter pylori* infection. Following RYGB, endoscopic surveillance of the remnant stomach becomes technically challenging, making routine cancer screening difficult. To address this issue, alternative approaches that avoid remnant stomach formation, such as sleeve plus procedures,^9)^ are being explored in Japan.

Kasama et al. developed DJB-SG to solve the problem of the remnant stomach and reported its safety and effectiveness.^1,10–12)^ However, DJB-SG poses technical challenges primarily due to the need for duodenal transection and complete intracorporeal anastomosis. In fact, DJB-SG has been performed only about one-tenth as often as SG over the same observation period and is limited to a few institutes. Since 2014, the number of DJB-SG procedures has decreased both in absolute terms and as a percentage of total procedures performed in Japan, coinciding with the inclusion of SG under national health insurance coverage.^13)^

In contrast, PJB-SG, originally reported in 2006 by Alamo et al. as sleeve gastrectomy with jejunal bypass (SGJB), avoids duodenal transection, simplifying the procedure by requiring only 1 jejunal bypass.^4,5)^ The initial development of the SGJB procedure was driven by the need to overcome the technical complexity of RYGB while achieving comparable therapeutic outcomes.

The most significant procedural difference between DJB-SG and PJB-SG is the presence or absence of duodenal transection. A potential consequence of duodenal transection is the development of complications, including the occurrence of a duodenal stump leak and disruption to endoscopic access to the bile and pancreatic ducts.

Duodenal stump leak may result in peritonitis, aneurysm, and the formation of intraperitoneal abscess due to the outflow of bile and pancreatic juice. Furthermore, the transection may also result in the development of an intractable fistula. A paucity of studies has specifically analyzed duodenal stump leaks in MBS that, akin to DJB-SG, engender a duodenal stump. Such leaks have been observed in biliopancreatic diversion with duodenal switch (BPD-DS) and single-anastomosis duodenoileal bypass with sleeve. Surve et al. reported a duodenal stump leak in 1 of 62 BPD-DS cases (1.6%).^14)^ The occurrence of other reports is inconsistent, resulting in uncertainty of the precise incidence. Baltasar et al. even described a fatal case,^15)^ suggesting that, although rare, duodenal stump leak is a complication that should not be underestimated.

Focusing on the necessity of accessing the bile and pancreatic ducts, a long-term cohort study in Norway provides useful information. According to a report by Belgau et al., the probability of requiring endoscopic retrograde cholangiopancreatography (ERCP) or surgical intervention after MBS appears to be approximately 1%.^16)^ Endoscopic access to the biliary tract is usually difficult after surgeries such as RYGB, so laparoscopy-assisted ERCP (LA-ERCP) or balloon enteroscopy–assisted ERCP (BEA-ERCP) may be necessary.^17)^ Although LA-ERCP has a high success rate, it is considered high risk due to the need for surgical intervention.^18)^ PJB-SG, on the other hand, allows access to the bile and pancreatic ducts via normal endoscopy, thus eliminating the need for procedures such as LA-ERCP and/or BEA-ERCP.

Furthermore, a jejunojejunal anastomosis is more technically simple when compared with a duodenojejunal anastomosis. The reduction in surgical complexity may make PJB-SG more accessible to a wider range of surgeons and facilitate its widespread adoption. The same single-anastomosis bypass technique without duodenal transection, termed single anastomosis sleeve jejunal bypass using the Billroth II technique, has also been reported.^19)^ Our rationale for selecting PJB-SG stems from the potential risk of future gastric carcinogenesis attributable to bile inflow into the stomach and the possibility of developing reflux esophagitis.

Although no long-term comparative studies between PJB-SG and SG have been reported, short-term outcomes suggest that PJB-SG may offer superior efficacy for T2DM. Seo and Ryu reported outcomes up to 12 months postoperatively, focusing on BMI and glycemic control in patients undergoing standalone SG and PJB-SG at a university hospital in South Korea.^20)^ According to their study, the type of surgical procedure was significantly associated with greater reductions in HbA1c levels at 6 and 12 months after surgery (*p* = 0.004 and 0.002, respectively), although no significant difference in BMI was observed between the 2 procedures. In contrast, Lin et al. reported a comparative analysis of SG plus jejunojejunal bypass (SG + JJB), a procedure conceptually similar to PJB-SG, versus SG alone and RYGB at a university hospital in China.^21)^ The findings indicated that SG + JJB resulted in enhanced weight reduction at 1 year postoperatively in comparison to SG alone (%TWL 38.8% vs. 35.0%, *p* = 0.011) while concurrently leading to a reduced incidence of nutritional complications, including hypoalbuminemia, vitamin deficiencies, and diarrhea, when compared with RYGB. Contrary to the conclusions reported by Seo and Ryu,^20)^ the observed additional effect on glycemic control was not detected in the SG + JJB group in comparison with the SG group.

**Fig. 4:**
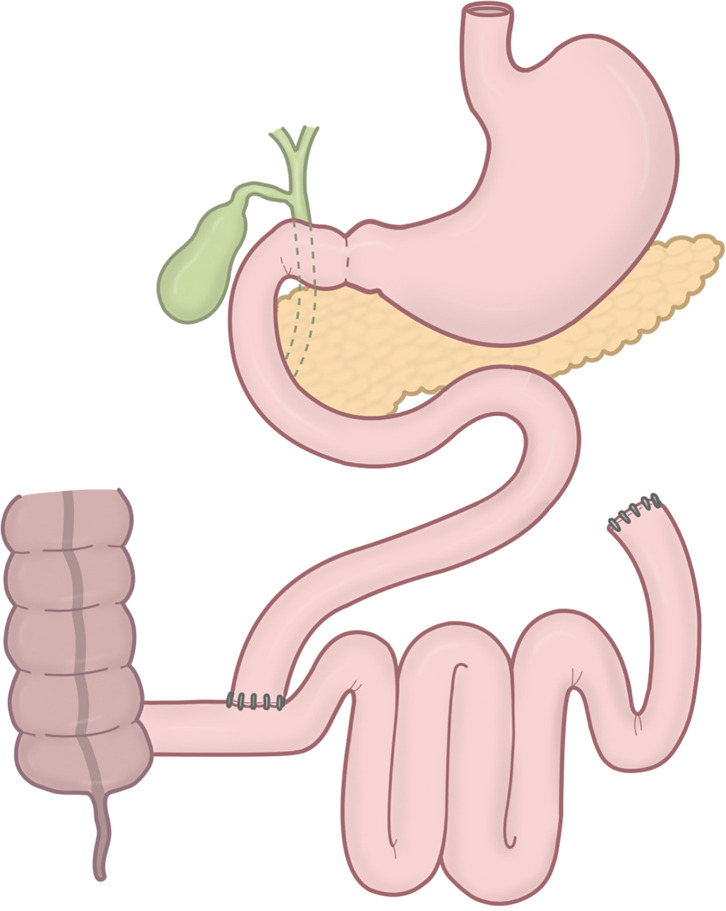
A schematic of JIB. In JIB, the proximal 35 cm of the jejunum is anastomosed to the distal 10 cm of the terminal ileum, bypassing over 90% of the small intestine without performing a gastrectomy. JIB, jejunoileal bypass

Regarding concerns about PJB, such as malabsorption and bacterial overgrowth in the bypassed small bowel, historical experience with jejunoileal bypass (JIB) in the 1960s and 1970s provides important lessons.^22)^ In JIB, the proximal 35 cm of the jejunum was anastomosed to the distal 10 cm of the terminal ileum, bypassing over 90% of the small intestine without performing a gastrectomy (**Fig. 4**). Although JIB achieved significant weight loss, it was frequently associated with severe malnutrition, liver dysfunction, and diarrhea, ultimately resulting in its abandonment. In contrast, PJB-SG preserves a significantly longer functional small intestine, ensuring adequate nutrient absorption while still delivering metabolic benefits in combination with SG. According to Sepúlveda et al., no cases of bacterial overgrowth or liver cirrhosis have been reported following PJB-SG, further supporting its safety profile.^23)^ All reported cases of conversion surgery after PJB-SG were due to gastroesophageal reflux disease associated with SG, rather than complications directly related to the PJB itself. In cases of extreme malnutrition, diarrhea, or blind-loop syndrome after PJB-SG, similar to complications following JIB, the bypassed intestinal segment can be reintegrated to prolong the length of the small intestine involved in digestion and absorption, thereby resolving such complications.

Patients who have undergone MBS may require revision surgery due to postoperative complications, including severe reflux esophagitis, inadequate weight loss, and/or poor glycemic control. In such cases, PJB-SG can achieve a revision to RYGB by utilizing the bypassed intestinal segment as an alimentary limb.

The main limitation of this report is its focus on the preliminary outcomes of a single case within a single institution. To confirm the long-term efficacy and safety of PJB-SG, further accumulation of cases is necessary.

## CONCLUSIONS

We present the first reported case of PJB-SG in Japan with a favorable postoperative course.

Future studies should evaluate the safety and efficacy of PJB-SG in the Japanese population to establish its role as a feasible option for MBS.
